# Synthesis of different crystallographic FeOOH catalysts for peroxymonosulfate activation towards organic matter degradation†

**DOI:** 10.1039/c7ra12615h

**Published:** 2018-02-14

**Authors:** Junyu Fan, Zhiwei Zhao, Zhaoxia Ding, Jie Liu

**Affiliations:** Department of Military Facilities, Army Logistics University Chongqing 401311 China liujiely@hotmail.com + 86 23 8673 0631; Key Laboratory of the Three Gorges Reservoir Region's Eco-Environment, State Ministry of Education, Chongqing University Chongqing 400045 China zhaozhiweihit@gmail.com

## Abstract

In this study, different crystalline structures of FeOOH have been prepared. α-FeOOH was synthesized through a hydrothermal method, whereas β-FeOOH was synthesized *via* a direct hydrolysis method. Moreover, γ- and δ-FeOOH were prepared by precipitation methods through slow and quick oxidation, respectively. On this basis, their crystal structure, morphology, and surface area were measured. Then, all the synthesized materials were applied to activate peroxymonosulfate (PMS) to generate sulfate radicals (SO_4_^−^˙) for acid orange 7(AO7) degradation. Compared with α-FeOOH, β-FeOOH, and γ-FeOOH, δ-FeOOH showed more efficient decolorization of AO7 in the catalytic system because of its abundant surface area and crystalline structure. The effects of several parameters in the δ-FeOOH/PMS/AO7 system were investigated. The results show that the initial pH, which is related to the features of surface hydroxyl groups, is the decisive factor, and excellent catalytic activity is maintained in the pH range 5–8. The increase of catalyst dosage and appropriate increase of PMS concentration contributed to promote the degradation effect. However, self-quenching was observed in a high PMS concentration system. Moreover, δ-FeOOH was stable after six consecutive cycles, and the leaching of iron ions was negligible. According to the quenching test and electron spin resonance analysis, both SO_4_^−^˙ and ˙OH were the dominant radicals for AO7 degradation.

## Abstract 2

In this study, different crystalline structures of FeOOH have been prepared.

## Graphical abstract

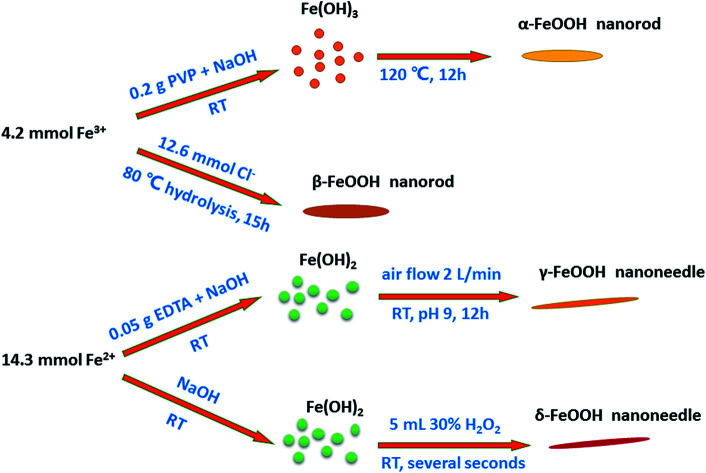

## 1. Introduction

In recent years, it has been proven that the radicals produced by the advanced oxidation process (AOP) can effectively attack the chromophoric group of the dye and make the dye mineralize completely. The common radicals used in wastewater treatment are hydroxyl radical (˙OH) and sulfate radical (SO_4_^−^˙).

The Fenton method, which relies on the hydroxyl radical, is the most widely used AOP. Compared to the traditional homogeneous Fenton method, heterogeneous Fenton method is more popular because it does not cause secondary pollution. Among all kinds of heterogeneous Fenton catalysts, iron-based materials, including Fe_2_O_3_,^1^ Fe_3_O_4_,^2^ MnFe_2_O_4_,^3^ and FeOOH,^4^ have always been the focus due to their wide sources, high performance, and low cost.

As an eco-friendly iron-based material with different crystalline structures (α-, β-, γ-, and δ-), hydroxyl iron oxide (FeOOH) has been reported to be used in different heterogeneous catalysis systems to remove dyes and other refractory contaminants from aqueous solutions. Silva *et al.* used δ-FeOOH as a catalyst to degrade rhodamine B in a photo-Fenton system.^5^ Wang *et al.* explored the activation effect of H_2_O_2_ to remove phenol by α-FeOOH/rGO composite materials.^6^ Zhang *et al.* prepared SBC@β-FeOOH composites in a heterogeneous Fenton-like reaction to remove doxycycline.^7^ Sheydaei *et al.* made reactive orange 29 as the target pollutant to explore the sonocatalytic decolorization of textile wastewater by γ-FeOOH nanoparticles.^8^ Moreover, FeOOH could effectively promote the generation of ˙OH in the presence of ozone.^9^

The sulfate radical mainly obtained by activating peroxymonosulfate and persulfate has the great advantage of its stabile oxidation reduction potential (2.01 eV at pH 7 and 1.96 eV at pH 4).^10–12^ Unlike persulfate, which requires other auxiliary methods (ultraviolet, ultrasound, and microwave), peroxymonosulfate (PMS) is more easily activated in a heterogeneous system in a neutral medium, especially by iron-based catalysts such as α-Fe_2_O_3_,^13,14^ Fe_3_O_4_,^14,15^ MnFe_2_O_4_,^16^ and Fe(0).^17^ However, no study has been reported on the activation of PMS with FeOOH to produce a sulfate radical. On the other hand, based on the existing literature, there are differences in the efficiencies of degradation when FeOOH with different structures is used.^5,8^ However, the influence of their different crystal structures on the degradation process has been rarely analyzed in detail.

In the present study, FeOOH nanoparticles with different crystal structures (α-, β-, γ-, and δ-) were synthesized and characterized. Then, the obtained solids were used as PMS activators for the first time to degrade acid orange 7 (AO7), a carcinogenic azo dye. After the activation effect was estimated, the catalytic mechanism was proposed according to the results.

## 2. Experimental

### 2.1. Materials

Ferric nitrate (Fe(NO_3_)_3_·9H_2_O), ferrous sulfate (FeSO_4_·7H_2_O), polyvinylpyrrolidone (PVP K30), ethylenediaminetetraacetic acid disodium salt (EDTA), urea, hydrochloric acid (HCl), sodium hydroxide (NaOH), hydrogen peroxide solution (30%), and ammonia solution (30%) were purchased from Sinopharm Chemical Reagent Co., Ltd (Shanghai China). Acid orange 7 (AO7) anhydrous ethanol, tertiary butyl alcohol (TBA), and oxone (PMS, KHSO_5_·0.5KHSO_4_·0.5K_2_SO_4_) were purchased from Aladdin Industrial Co. Ltd. (Shanghai, China). All the solutions were prepared with deionized water.

### 2.2. Synthesis of catalysts

**Fig. 1:**
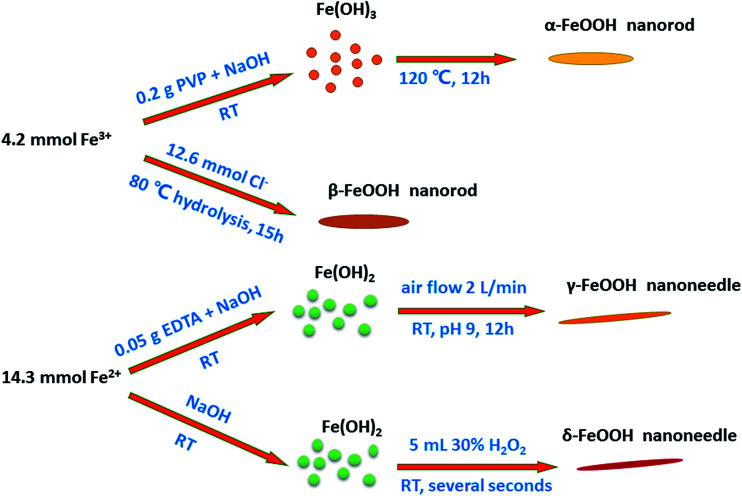
Proposed schematic of the synthesis of different crystal structures of FeOOH nanorods/needles.

Preparation of all the crystal structures of catalysts was directly adopted from previously reported methods. The schematic of the preparation of each crystal type of FeOOH is shown on Fig. 1, and the detailed synthesis methods have been described hereinafter.

#### 2.2.1. Synthesis of the α-FeOOH catalyst

α-FeOOH can be synthesized from either Fe(iii) or Fe(ii) systems. Due to the need for careful control to prevent other oxidation products for Fe(ii) systems, the Fe(iii) system is recommended. In the Fe(iii) system, α-FeOOH can be formed over a wide pH range, either in acidic or alkaline media.^18^ Because of the rapid formation of precipitates in the alkaline media, it is generally used. In the alkaline media, synthesis involves holding freshly prepared ferrihydrite, which is the precursor of α-FeOOH, at pH > 12 for several days.^18^ Recently, to decrease the aging time, a hydrothermal method was adopted.^19,20^

Briefly, 0.2 g of PVP (used to improve the dispersivity of particles in the hydrothermal process^20,21^) was added to a 25 mL solution containing 1.7 g Fe(NO_3_)_3_·9H_2_O. Then, a 9 mL solution of NaOH (5 M) was added to the mixture under vigorous stirring. After 3 h, stirring was stopped, and the stable suspension was transferred into a 100 mL Teflon-lined stainless-steel autoclave that was maintained at 120 °C for 12 h.

When cooled down to environment temperature, the sediment was washed three times with deionized water and anhydrous ethanol alternately. The product was obtained after drying at 60 °C for 6 h.

#### 2.2.2. Synthesis of the β-FeOOH catalyst

β-FeOOH was prepared by the hydrolysis of a Fe(iii) chloride solution. In the synthesis process, chloride ion occupies the 0.5 × 0.5 nm^2^ interstices in the tunnels of the structure and appears to direct this structure and stabilize it. β-FeOOH cannot be prepared at pH > 5 because OH^−^ ion is far more competitive than chloride ion for structural sites.^18^

The β-FeOOH catalyst was synthesized by a direct hydrolysis method. The typical synthesis was as follows: 1.62 g of FeCl_3_·6H_2_O was dissolved in 150 mL of deionized water, and the solution was continuously stirred for 15 h at 80 °C. Then, a suspension was obtained. After centrifugation, it was washed 3 times with deionized water and dried at 60 °C for 6 h to obtain the product.

#### 2.2.3. Synthesis of the γ-FeOOH catalyst

γ-FeOOH was conveniently synthesized by oxidizing an Fe^2+^-containing solution at a pH close to neutral, and the pH needed to be maintained during the entire process to ensure that protons could be produced:^18^4Fe^2+^ + O_2_ + 6H_2_O → 4γ-FeOOH + 8H^+^

The γ-FeOOH catalyst was synthesized by an easy precipitation method. Herein, 0.05 g of EDTA (used to ensure the purity of γ-crystals and inhibit the generation of α-FeOOH^22,23^) was added to a 100 mL solution containing 3.97 g of FeSO_4_·7H_2_O. Then, the pH of the solution was adjusted to 6.5–7.5 by adding NaOH dropwise under vigorous stirring. Moreover, the bubbling started with an air rate of 2 L min^−1^ for 12 h. The solid precipitate was obtained by centrifugation and washing 3 times with deionized water. The final product was obtained after drying at 60 °C for 6 h.

#### 2.2.4. Synthesis of the δ-FeOOH catalyst

δ-FeOOH is a ferrimagnetic mineral that is usually produced by the H_2_O_2_ oxidation of Fe(OH)_2_ at a high pH. Very rapid oxidation is essential for the formation of δ-FeOOH because if the oxidation rate is lowered, γ-FeOOH or Fe_3_O_4_ may form.^18^

The δ-FeOOH catalyst was synthesized by a modified precipitation method. Typically, 3.97 g of FeSO_4_·7H_2_O was dissolved in 100 mL of deionized water. Then, a 20 mL solution of NaOH (5 M) was immediately added to the metal ion solution under vigorous stirring. After this, 5 mL of 30% H_2_O_2_ was injected into it to provide the necessary rapid oxidation for the formation of crystalline structures.^24–26^ After 1 min, the precipitate was centrifuged and washed 3 times with deionized water. The final product was obtained after drying at 60 °C for 6 h.

### 2.3. Characterization of the catalysts

Transmission electron microscopy (TEM) and high-resolution TEM (HRTEM) were employed to investigate the morphology and microstructure of the catalysts (JEOL, JEM-2100F, Japan, working at 200 kV). The X-ray powder diffraction (XRD) pattern was employed to determine the crystallinity of the catalysts (Empyrean, Manalytical, the Netherlands) at 40 kV and 30 mA over the 2*θ* range 10–80°. X-ray photoelectron spectroscopy (XPS) experiments were used to identify the valency of elements (Escalab 250Xi, Thermo Fisher Scientific, US). The Brunauer–Emmett–Teller (BET) method was used to measure the specific surface area and the pore structure of the catalysts (ASAP 2020, Quantachrome, US, performed at 77 K). Moreover, the isoelectric point (pH_pzc_) was measured by zetasizer (Malvern U.K.). Thermogravimetric analysis (TGA) was carried out using a TGA/DSC1 STAR thermogravimetric analyzer from 50 to 400 °C at a heating rate of 3 °C min^−1^ in a N_2_ flow.

### 2.4. Catalytic experiments

Catalytic experiments were conducted in common 250 mL conical flasks at 25 °C with 120 rpm. The catalyst suspensions were prepared by dispersing the catalysts in flasks with deionized water under ultrasonication (Xinzhing Co., Ltd, China). Then, some AO7 solution (1.0 g L^−1^) and oxone solution (0.1 M) were added to the mixture. Moreover, the pH of the reaction mixture was adjusted using 0.1 M HCl and 0.1 M NaOH. After all the abovementioned steps, the total volume of each suspension was adjusted to 100 mL with deionized water.

The AO7 concentration was analyzed using a spectrophotometer (T9, Persee, China) at 484 nm immediately after the suspensions were filtered through 0.22 μm hydrophilic polyethersulfone membranes (Huaxia Co. Ltd, China). The concentration of iron ion was analyzed by inductively coupled plasma atomic emission spectroscopy (ICP-MS, Elemental Scientific, US). Moreover, the generated radical products were analyzed *via* competitive dynamics by bringing in a radical scavenger and detected by an electron spin resonance spectrometer (ESR) (Albutran, AXM-09, US).

## 3. Results and discussion

### 3.1. Characterization of FeOOH

**Fig. 2:**
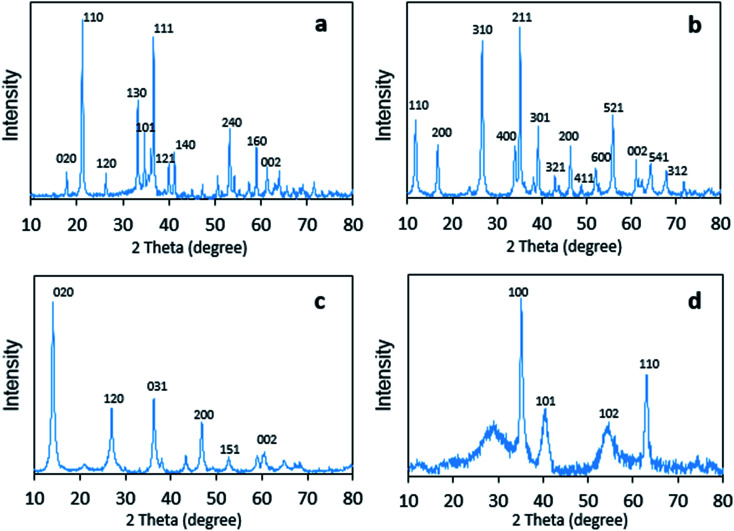
Powder X-ray diffraction patterns of (a) α-FeOOH, (b) β-FeOOH, (c) γ-FeOOH, and (d) δ-FeOOH.

The crystal structures of the four FeOOH catalysts were analyzed by wide-angle XRD patterns, as shown in Fig. 2. It could be seen that all of them corresponded to the standardized structures of JCPDS, and no other phases were found. For α-FeOOH, sharp diffraction peaks appeared at 17.7°, 21.1°, 26.3°, 33.2°, 34.6°, 36.6°, 39.9°, 41.1°, 53.2°, 59.0°, and 61.2°, which agreed well with those of goethite (JCPDS 29-0713). In addition, the pattern of β-FeOOH showed peaks, which appeared mainly at 11.9°, 16.8°, 26.7°, 34.0°, 35.2°, 39.2°, 43.1°, 46.5°, 52.1°, 55.9°, 61.3°, 64.2°, and 67.9°, that were consistent with those of akaganeite (JCPDS 75-1549). The crystal configuration of γ-FeOOH was similar to that of lepidocrocite (JCPDS 08-0098), which had sharp diffraction peaks appearing at 14.2°, 27.2°, 36.5°, 46.9°, 53.2°, and 59.0°. In addition, δ-FeOOH, consistent with feroxyhyte (JCPDS 13-0087), possessed sharp diffraction peaks at 35.2°, 40.6°, 54.5°, and 63.1°.

**Fig. 3:**
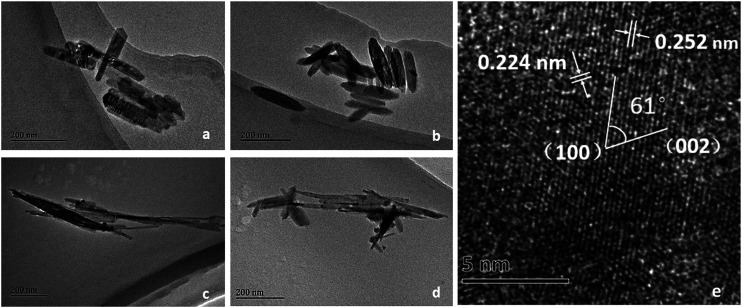
TEM and HRTEM images of (a) α-FeOOH, (b) β-FeOOH, (c) γ-FeOOH, and (d) δ-FeOOH; (e) HRTEM image of δ-FeOOH.

The morphologies and microstructures were observed by TEM and HRTEM. As can be seen in Fig. 3(a), α-FeOOH exhibited the morphology of short irregular nanorods. On the other hand, the morphology of β-FeOOH was a regular spindle-shape with a length of 200 nm, as shown in Fig. 3(b). The morphology of γ-FeOOH shown in Fig. 3(c) was needle-like particles with most widths between 20 and 30 nm. δ-FeOOH had a completely similar microstructure (Fig. 3(d)) to γ-FeOOH, which also exhibited needle-like particles. Furthermore, as observed in the HRTEM image (Fig. 3(e)), the lattice fringe spacings of feroxyhyte-structure of δ-FeOOH were about 0.29 nm for the (100) plane and 0.23 nm for the (002) plane.

**Fig. 4:**
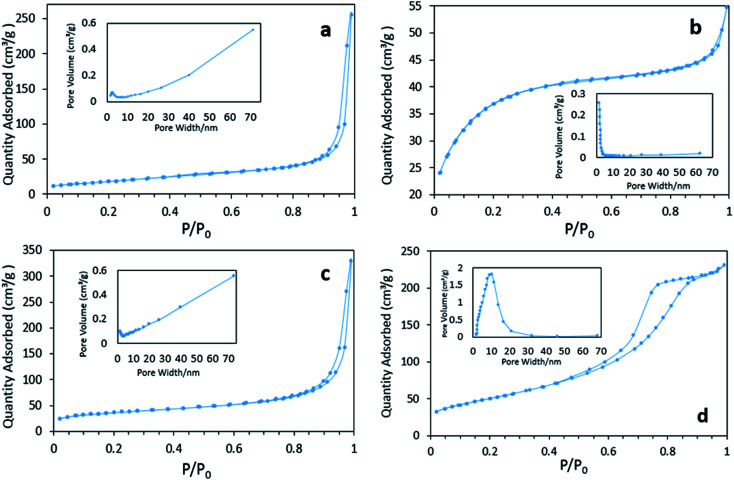
The N_2_ absorption/desorption isotherm curves and pore size distributions of catalysts: (a) α-FeOOH, (b) β-FeOOH, (c) γ-FeOOH, and (d) δ-FeOOH.

Fig. 4 displays the N_2_ physisorption isotherm and pore diameter distribution of various FeOOH solids. Both the BET surface areas and average pore diameters are listed in Table 1. It can be seen that δ-FeOOH has highest BET surface area, whereas α-FeOOH displays lowest BET surface area. According to the pore diameter distribution results, macropores are dominant in α- and γ-FeOOH because of the formation of a loose structure intermediate with the quick-added precipitator.^27^ On the other hand, β-FeOOH is rich in micropores, which are induced by the slow process of hydrolysis.^28^ It is interesting to note the highly centralized distribution of mesoporous structures of δ-FeOOH. This can be further confirmed by the type IV isotherm of its adsorption–desorption curve.^29,30^ The abundant mesopores may be related to the microbubbles produced by H_2_O_2_ decomposition during the preparation. The pH_pzc_ values of the four materials (α-, β-, γ-, and δ-FeOOH) were 6.82, 7.31, 6.38, and 5.84, which were roughly consistent with the values reported in literature.^31^ All the results may be associated with different crystal types.

**Table 1:** Physicochemical properties of the four different FeOOH catalysts

The crystal type	*S*_BET_ (m^2^ g^−1^)	Volume of pores (cm^3^ g^−1^)	Average pore width (nm)	pH_pzc_
α-FeOOH	64.1	0.50	21.73	6.82
β-FeOOH	133.6	0.06	3.32	7.31
γ-FeOOH	131.4	0.39	18.80	6.38
δ-FeOOH	179.7	1.24	6.46	5.84

### 3.2. Catalytic activity of different FeOOH catalysts

**Fig. 5:**
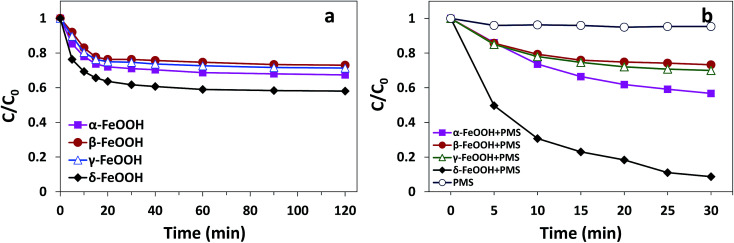
(a) The efficiencies of AO7 adsorption by different crystal types of FeOOH. Conditions: [AO7] = 50 mg L^−1^, adsorbent = 0.3 g L^−1^, pH 5 and *t* = 25 ± 1 °C. (b) The degradation efficiency of AO7 in FeOOH/PMS systems. Conditions: [AO7] = 50 mg L^−1^, PMS : AO7 (mol) = 20 : 1, catalyst = 0.3 g L^−1^, pH 5 and *t* = 25 ± 1 °C.

The adsorption and degradation of AO7 on various FeOOH solids are displayed in Fig. 5. It can be seen that all four materials demonstrated adsorption efficiencies for AO7. However, mere adsorption is highly limited to the removal of pollutants. As can be seen in Fig. 5(a), within 120 min, α-FeOOH, β-FeOOH, and γ-FeOOH have similar adsorption efficiencies (27% for α-, 24.4% for β-, and 23.7% for γ-), whereas δ-FeOOH has the highest adsorption efficiency (39.7%).

Moreover, similar trends could be observed for degrees of discoloration when different forms of FeOOH were combined with PMS. It can be seen from Fig. 5(b) that the decolorization efficiency is as high as 91.4% in the δ-FeOOH/PMS system as compared to that of the other three groups (42% for α-FeOOH/PMS, 24.9% for β-FeOOH/PMS, and 29.5% for γ-FeOOH/PMS) after 30 min of the catalytic reaction. In addition, simple addition of PMS to the AO7 solution resulted in almost no discoloration (4.7%). Thus, it is obvious that α-FeOOH and δ-FeOOH have activation capacities for PMS, whereas β-FeOOH and γ-FeOOH does not, and the activation capacity of δ-FeOOH is much stronger than that of α-FeOOH.

Ji *et al.* have found that the higher PMS activation capacity of the prepared porous α-Fe_2_O_3_ as compared to that of the commercial α-Fe_2_O_3_ may be attributed to the larger surface area of the former.^32^ Saputra *et al.* found that α-MnO_2_ exhibited higher adsorption due to its larger surface area, which promoted the reaction between the sulfate radical and phenol.^16^ Wang *et al.* reported that meso-CuFe_2_O_4_ with a high surface area displayed a higher catalytic activity than commercial CuFe_2_O_4_.^33^ Among the four FeOOH solids, δ-FeOOH has more surface area than the other three, which provides more active sites in the adsorption and heterogeneous catalytic reaction.

**Fig. 6:**
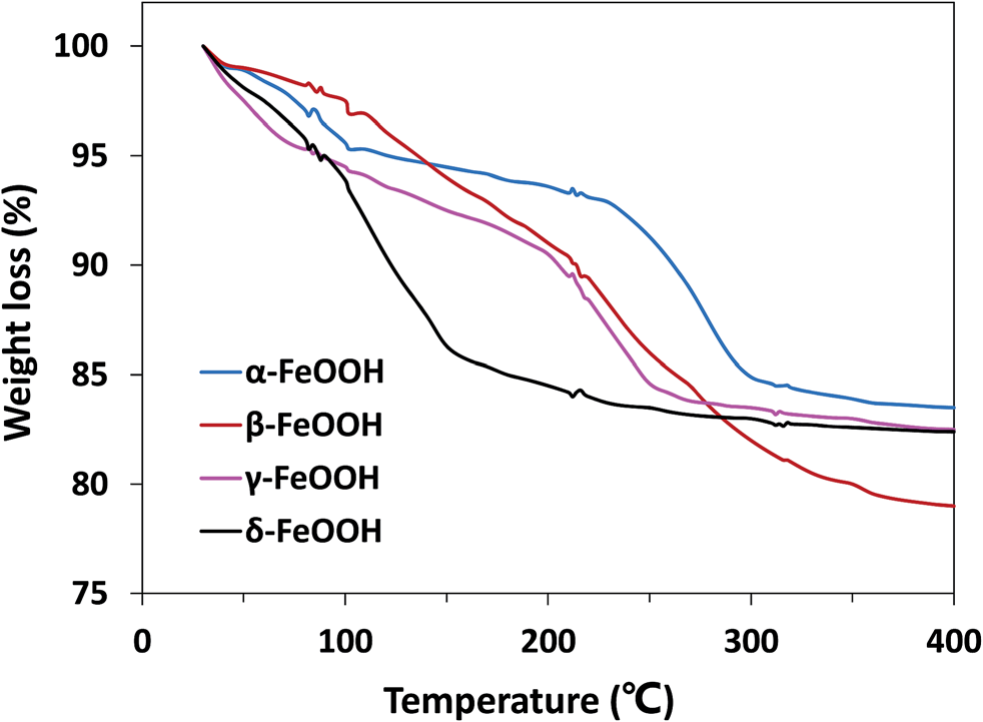
TGA curves for α-FeOOH, β-FeOOH, γ-FeOOH, and δ-FeOOH compounds (heating rate: 3 °C min^−1^).

Moreover, the effect of the crystalline structures of the catalysts cannot be ignored. The atomic configurations of all four crystal structures of FeOOH polymorphs are given in Fig. S1.† α-FeOOH has the same structure as diaspore (α-AlOOH), a typical orthorhombic system. Fe^3+^ in crystals is hexagonal close packed to make [FeO_3_(OH)_3_] an octahedral structure with anions around.^34^ β-FeOOH belongs to the tetragonal system with a (2 × 2) tunnel structure.^18^ γ- and δ-FeOOH are layered crystal structures constituted by octahedral [FeO_6_], belonging to the orthorhombic system and hexagonal system, respectively.^35^ Because of the abovementioned different structures, the bound water has different locations in the crystal; this leads to hydration of different strengths. Therefore, TGA analysis was performed to demonstrate the location of the structural water in different FeOOH solids. As shown in Fig. 6, δ-FeOOH exhibited highest weight loss of surface adsorbed water from room temperature to 150 °C. Previous studies have reported that water adsorbed on the surface of catalysts can enhance the catalytic rate.^36,37^ Therefore, the more amount of surface water may induce a higher catalytic activity. δ-FeOOH showed the highest amount of adsorbed water loss from TGA, which was coincident with the result of its highest catalytic efficiency. Due to its excellent specific surface area and crystalline structure, δ-FeOOH owns most active sites among the four solids, leading to the best catalytic activity. However, α-FeOOH with a lower surface area presented a higher catalytic efficiency than γ-FeOOH although their hydrations were similar. This could be attributed to the relatively weak surface FeO–H bonds of α-FeOOH that seemed to favor the interaction of surface hydroxyl groups with HSO_5_^−^.^31^ The relatively weak surface FeO–H bonds of the hydroxylated α-FeOOH lead to a high affinity of its electrophilic H; this makes the surface OH-PMS combination easy. Therefore, the surface hydroxyl groups of α-FeOOH exhibited higher catalytic activity than γ-FeOOH in promoting PMS decomposition.

### 3.3. Effect of reaction conditions on AO7 degradation

In the following experiments, δ-FeOOH was mainly used as a heterogeneous catalyst to investigate the influence of various factors, including catalyst dosage, oxidant dosage, and pH, on the catalytic process.

#### 3.3.1. Effect of catalyst dosage

**Fig. 7:**
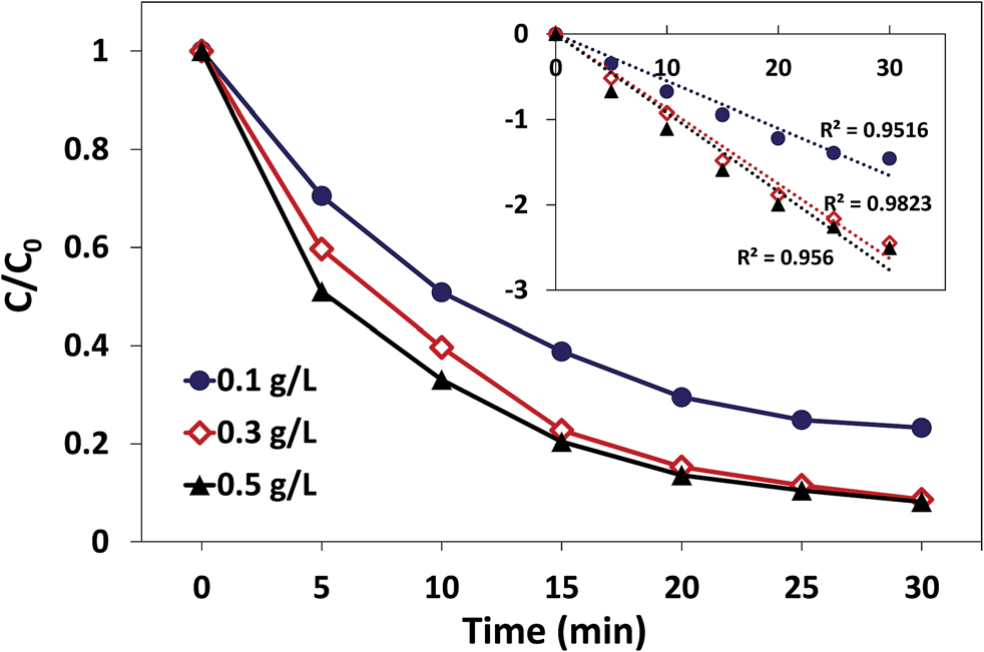
Effect of catalyst dosage on AO7 degradation in the δ-FeOOH/PMS process. Conditions: [AO7] = 50 mg L^−1^, PMS : AO7 (mol) = 20 : 1, pH 5 and *t* = 25 ± 1 °C.

The effect of catalyst dosage on AO7 degradation in the δ-FeOOH/PMS system is presented in Fig. 7. As displayed, the AO7 degradation under different catalyst dosages was consistently well-fitted by the pseudo-first-order kinetic model, whereas the amount of the catalyst had a significant influence on the AO7 degradation process. When the dosage of the catalyst increased from 0.1 g L^−1^ to 0.3 g L^−1^, the degradation rate increased from 0.055 min^−1^ to 0.088 min^−1^, and the decolorization efficiency was promoted from 76.8% to 91.4% in 30 min. The notable improvement might be attributed to more active sites provided by more catalysts such that more radicals could be produced in a short time.^38^ However, the decolorization efficiency only increased to 92.8%, and the degradation rate increased to 0.092 min^−1^ when the catalyst dosage was increased to 0.5 g L^−1^. This might be related to the insufficient concentration of PMS in the reaction systems with high catalyst dosages.

#### 3.3.2. Effect of the oxidant dosage

**Fig. 8:**
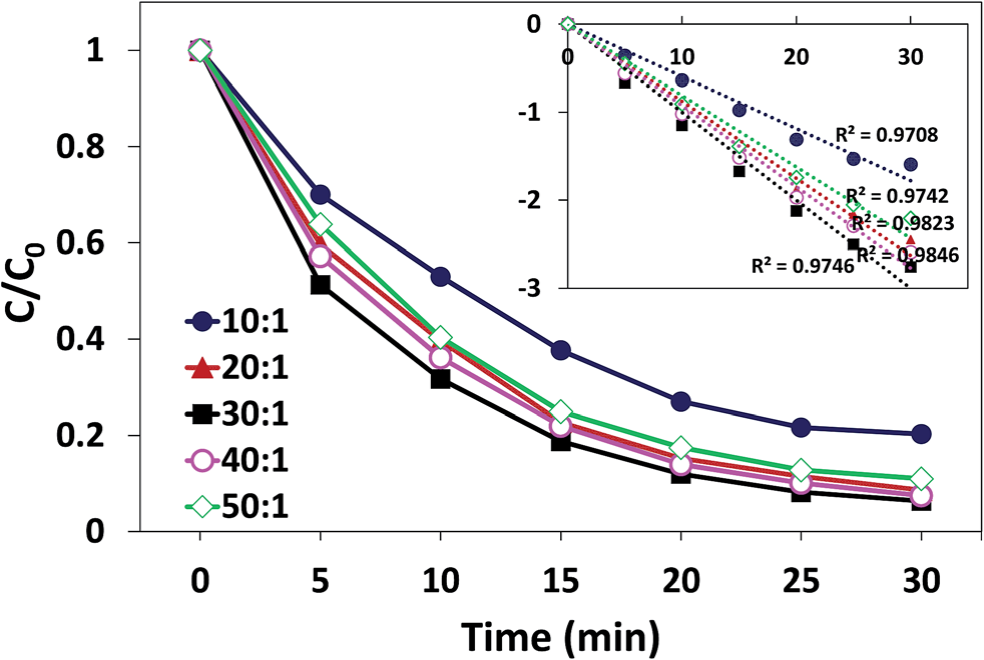
Effect of oxidant dosage (mole ratio of PMS : AO7) on AO7 degradation in the δ-FeOOH/PMS process. Conditions: [AO7] = 50 mg L^−1^, catalyst = 0.3 g L^−1^, pH 5, and *t* = 25 ± 1 °C.

Oxidant dosage can be represented by the mole ratio of oxidant (PMS) and substrate (AO7). The influence of the mole ratio of PMS/AO7 on degradation process is illustrated in Fig. 8. When the mole ratio of PMS/AO7 was changed from 10 : 1 to 30 : 1, the degradation rate constant rapidly increased from 0.063 to 0.099 min^−1^. However, when the mole ratio was increased from 30 : 1 to 50 : 1, the rate decreased from 0.099 to 0.093 min^−1^. This phenomenon is consistent with many other heterogeneous catalytic reactions for the activation of PMS.^39^ The increase in PMS concentration within a certain range is conducive for producing more free radicals to attack pollutants. However, too many unreacted PMS in the solution will quench the produced free radicals as shown in the following reaction:^40^

1 SO_4_^−^˙ + HSO_5_^−^ → SO_5_^−^˙ + HSO_4_^−^

2 ˙OH + HSO_5_^−^ → SO_5_^−^˙ + H_2_O

Moreover, the limited active sites of the catalyst hinder the increase in the degradation rate. On the other hand, the degradation efficiency of AO7 was 91.7% for the molar ratio of 20 : 1 and 93.6% for 30 : 1; thus, the optimal molar ratio was 20 : 1.

#### 3.3.3. Effect of initial pH

**Fig. 9:**
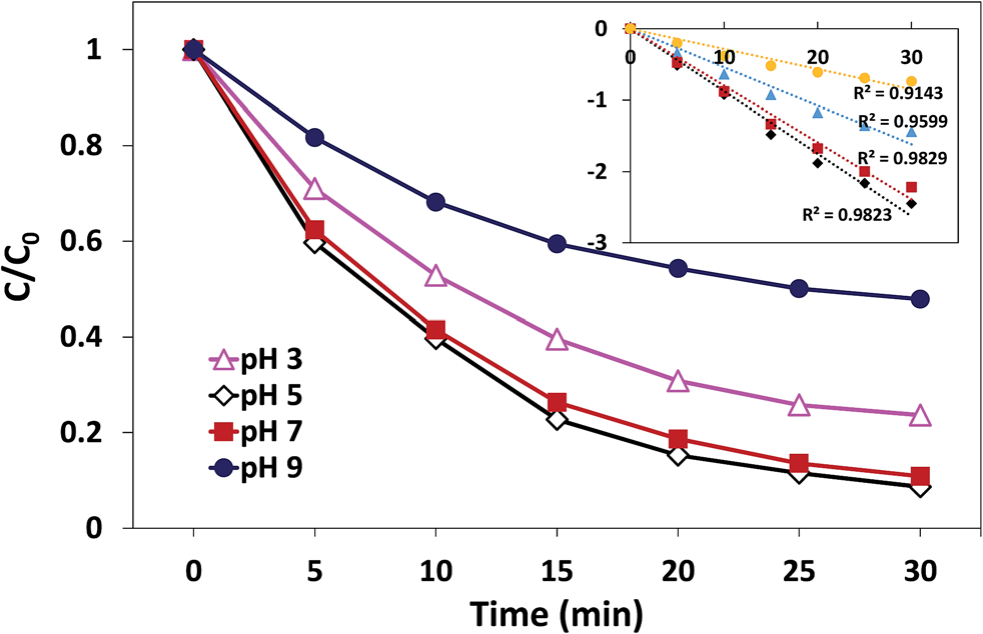
Effect of initial pH on AO7 degradation in the δ-FeOOH/PMS process. Conditions: [AO7] = 50 mg L^−1^, catalyst = 0.3 g L^−1^, PMS : AO7 = 20 : 1 and *t* = 25 ± 1 °C.

The effect of initial pH on the degradation process has been demonstrated in Fig. 9. Obviously, the effect of initial pH on catalysis is significant. The most efficient AO7 degradation occurred at pH 5. The degradation rate and decolorization efficiency of AO7 were 0.088 min^−1^ and 91.7%, respectively. When pH was reduced to 3, the degradation rate decreased to 0.054 min^−1^, and the decolorization efficiency was 79.3%. When the solution alkalinity was increased, the degradation rate started to decrease. The value was 0.080 min^−1^ at pH 7 (decolorization efficiency was 88.1%), whereas it underwent a sharp decrease to 0.028 min^−1^ at pH 9 (decolorization efficiency was 60%). These results may be related to the charge state of catalyst surface and the species of PMS in the aqueous solution.^23,41^ The pH_pzc_ of δ-FeOOH is 5.84. Most of the surface hydroxyl groups are at a neutral state when pH is close to pH_pzc_. When pH is far below or above the pH_pzc_, the surface will be charged as follows:^31^

3 Fe(iii)_surface_–OH + H^+^ → Fe(iii)_surface_–OH_2_^+^

4 Fe(iii)_surface_–OH + OH^−^ → Fe(iii)_surface_–O^−^ + H_2_O

Thus, when the pH is 3, the catalyst surface is highly protonated, which is unfavorable for the non-polar ends of the accessing organic matter. When pH was 5 and 7, both acid centers and alkaline centers existed on the surface of catalysts, which induced the organic matter to easily access the interface.^42^ On the other hand, since the catalyst surface is heavily negatively charged at pH 9, HSO_5_^−^ and organic matter hardly interact with the catalysts. Moreover, at pH 9, HSO_5_^−^ will further transform into SO_5_^2−^; thus, the electrostatic repulsion between the anion and catalysts becomes stronger.^23^ In addition, when solution pH exceeds 9, ˙OH would scavenge SO_4_^−^˙ and become the dominant active species,^31^ which possesses reduced oxidative capacity. These findings can explain the sharp decrease in the decolorization efficiency at pH 9.

### 3.4. Reusability and stability

Reusability is an important factor that evaluates the performance of the catalyst in practical applications.^43,44^ Therefore, successive experiments were conducted to explore the reusability of δ-FeOOH under the same conditions. After each trial, the used catalysts were obtained followed by washing with ethanol and deionized water, separation by centrifugation, and then drying at 60 °C. The catalysts were repeatedly used six times. Table 2 shows the decolorization efficiency of AO7 and the leached concentration of Fe^3+^ in solution at each catalyst cycle. After recycling for six times, the δ-FeOOH/PMS system could still maintain a high catalytic efficiency. The decolorization efficiency of AO7 only decreased from 91.7% to 84.2%. Furthermore, the leached concentration of Fe^3+^ in the solution after each reaction cycle was determined using ICP-MS. As can be seen from Table 2, the leaching of metal ions was always under 5 μg L^−1^. Both these results revealed the high stability of the catalysts, and the degradation reaction occurred at the interface of δ-FeOOH.^45^ In addition, the same successive experiments were carried out on the other three types of crystals, and the decolorization efficiency and the leached concentration of Fe^3+^ in each experiment are shown in the Tables S1 and S2,† respectively. Besides, the AO7 degradation in other similar systems has been listed as a comparison (Table 3).

**Table 2:** Decolorization efficiencies and leached metal ion amounts of δ-FeOOH during six consecutive cycles

Times reused	Decolorization efficiency (%)	Leached iron concentration (μg L^−1^)
1	91.7	4.48
2	91.2	4.02
3	88.1	3.22
4	87.2	2.85
5	86.3	2.33
6	84.2	2.01

**Table 3:** The AO7 degradation activity of δ-FeOOH/PMS as compared to that of similar systems

System	Reaction conditions							Degradation
	AO7 concentration	Oxidant concentration	Activation agent dose	Temperature (°C)	pH	Reaction time	Assistant	
δ-FeOOH/PMS	50 mg L^−1^	0.43 g L^−1^	0.3 g L^−1^	25	5.0	30 min	—	91.4%
Fe_3_O_4_/PMS^50^	0.06 mM	3 mM	0.4 g L^−1^	25	7.5	30 min	Ultrasound 0.2 kW	90%
S-Doped α-Fe_2_O_3_/H_2_O_2_ (ref. 58)	35 mg L^−1^	1.96 mM	0.1 g L^−1^	25	6.85	14 min	Halogen lamp 1 kW	95%
α-FeOOH^59^	20 mg L^−1^	—	0.075 g L^−1^	RT	7.0	120 min	UVA lamp	85%
rGO-Fe_3_O_4_/H_2_O_2_ (ref. 60)	20 mg L^−1^	22 mM	0.2 g L^−1^	25	3.0	60 min	—	85%
Nano-ZVI/PS^61^	0.2 mM	2 mM	0.3 g L^−1^	25	3.8	30 min	—	97.5%

**Fig. 10:**
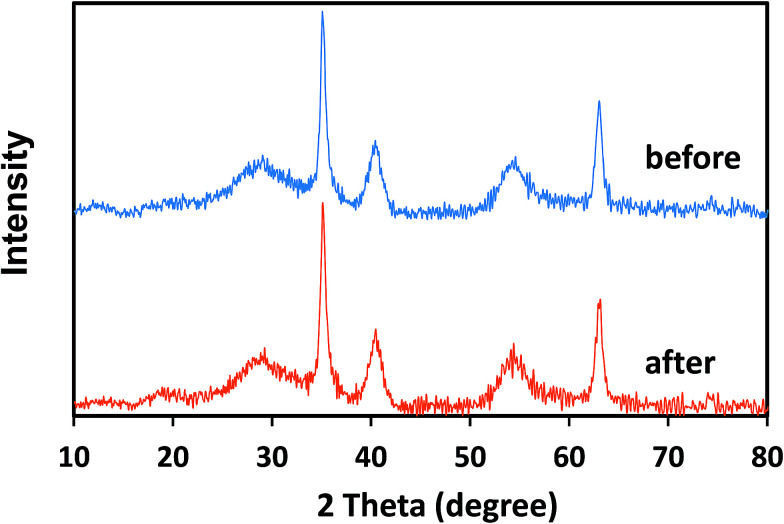
XRD patterns of synthesized δ-FeOOH before and after its use for 6 times in the PMS/δ-FeOOH/AO7 system.

The stability of the crystal structure of catalysts was determined *via* XRD analysis. As can be seen from Fig. 10, there are no obvious changes in the diffraction peaks of δ-FeOOH after six cycles as compared to those of freshly prepared δ-FeOOH. This result revealed the well stability of the crystal structures of δ-FeOOH in the PMS catalytic system. Moreover, the XRD patterns of other three crystal FeOOH are shown in Fig. S2,† and all the crystal structures do not change after the reaction. However, due to the relatively low catalytic efficiency, further studies on them were not carried out.

**Fig. 11:**
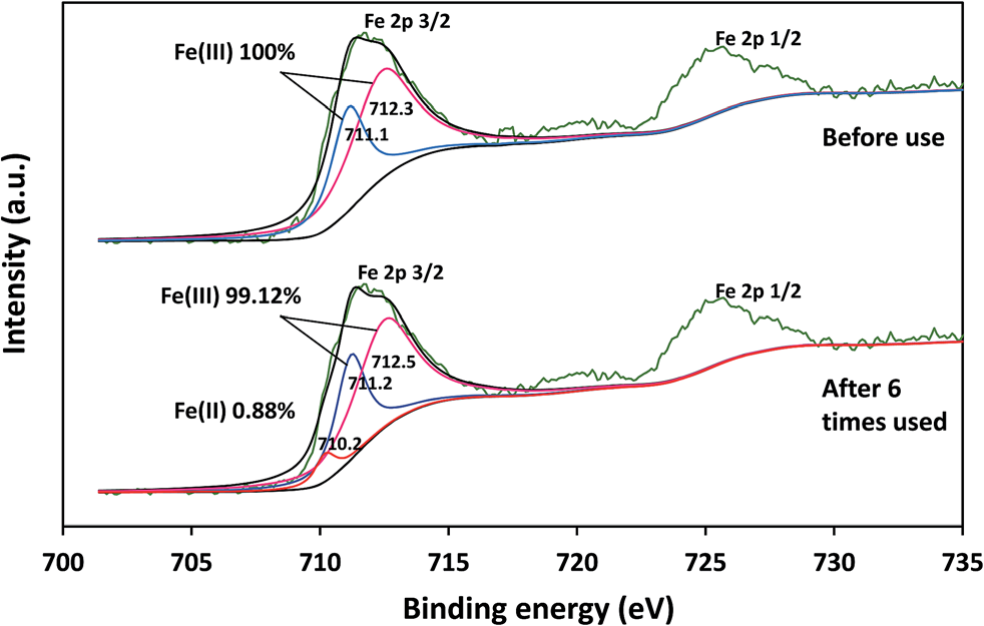
Fe 2p XPS spectrum of synthesized δ-FeOOH before and after its use for 6 times in the PMS/δ-FeOOH/AO7 system.

The elemental changes on the surface of catalysts before and after the reaction cycle could be confirmed *via* XPS analysis. As can be seen from Fig. 11, the Fe 2p_3/2_ peak was present at 711.0 eV for the fresh catalyst, whereas it was at 711.2 eV for the catalyst after six cycles. The appearance of 0.88% Fe(ii) in the catalysts after six cycles indicated the occurrence of the reduction process during the reaction. The results indicated that δ-FeOOH was suitable to be used as a PMS activator.

### 3.5. Reactive species

**Fig. 12:**
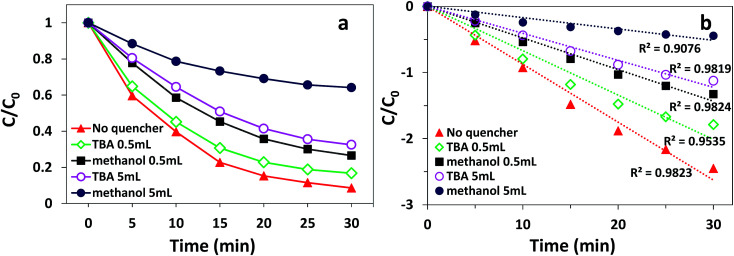
Radical quenching test on AO7 degradation in the δ-FeOOH/PMS process: (a) decolorization efficiency and (b) degradation rate fitted by the pseudo-first-order kinetic model. Conditions: [AO7] = 50 mg L^−1^, catalyst = 0.3 g L^−1^, PMS : AO7 = 20 : 1 and *t* = 25 ± 1 °C.

According to previous reports, PMS can produce multiple radicals such as SO_4_^−^˙, ˙OH, and SO_5_^−^˙.^46^ Among them, SO_5_^−^˙ cannot decolorize AO7 owing to its low oxidative potential.^47^ To explore which radical (SO_4_^−^˙ or ˙OH) played the major role in the degradation process of AO7, both ethanol and *tert*-butanol (TBA) were added to the solution as radical quenching agents. Ethanol can react at a high rate with both SO_4_^−^˙ and ˙OH (*k*_SO_4_^−^˙_ = 8.6 × 10^9^ M^−1^ s^−1^; *k*_˙OH_ = 6.4 × 10^9^ M^−1^ s^−1^). However, TBA can only react rapidly with ˙OH (*k*_˙OH_ = 3.8–7.6 × 10^8^ M^−1^ s^−1^; *k*_SO_4_^−^˙_ = 4–9.1 × 10^5^ M^−1^ s^−1^).^48^ The effect of different quenchers on the degradation of AO7 in the δ-FeOOH/PMS process is shown in Fig. 12. As presented, the decolorization efficiency was 91.7% in 30 min without any quenchers. When 0.5 mL ethanol was added, the decolorization efficiency decreased to 74.1%. When the amount of ethanol was increased to 5 mL, the decolorization efficiency sharply decreased to 35.9%. When TBA was used as a radical quencher, the decolorization efficiencies under the same conditions were 83.6% and 62.3%. The results show that the degradation of AO7 is a radical reaction, and both SO_4_^−^˙ and ˙OH are generated in δ-FeOOH/PMS to attack AO7.

**Fig. 13:**
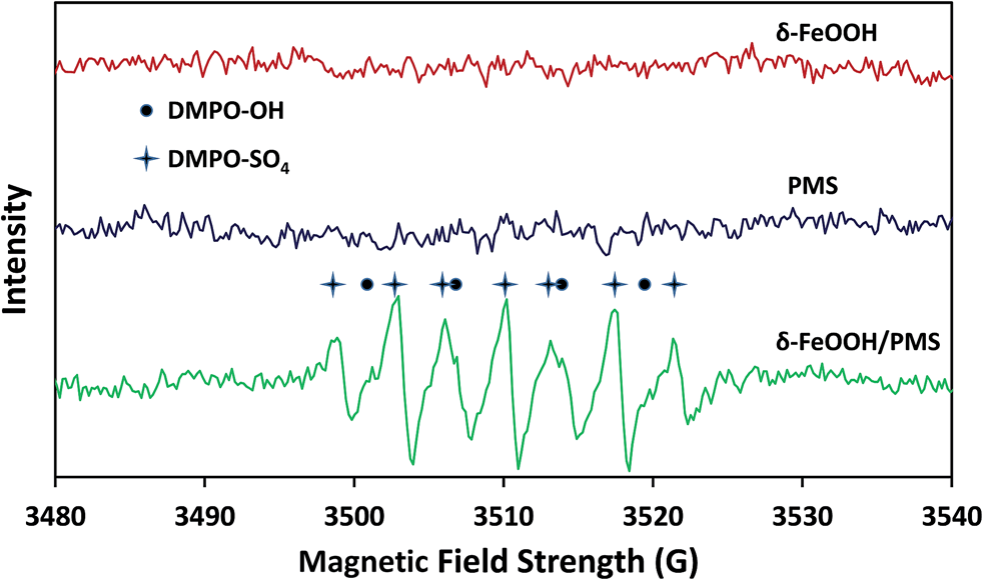
ESR spectra of the δ-FeOOH particles in deionized water, the PMS solution, and the δ-FeOOH particles in a PMS solution. The black dots represent DMPO-OH and the four-pointed stars represent DMPO-SO_4_. Conditions: δ-FeOOH = 0.3 g L^−1^, PMS = 5 mmol L^−1^, [DMPO]_0_ = 10 mmol L^−1^, pH 5 and *t* = 25 ± 1 °C. The settings for spectrometer: center field, 3510 G; sweep width, 120 G; frequency, 9.25 GHz; modulation frequency, 100 kHz; power, 20 mW.

To further strongly prove that both SO_4_^−^˙ and ˙OH were generated in the δ-FeOOH/PMS system, ESR tests were conducted to detect SO_4_^−^˙ and ˙OH during the catalytic process. DMPO was used as the spin-trapping agent, which formed complexes with SO_4_^−^˙ and ˙OH. Then, SO_4_^−^˙ and ˙OH could be detected by measuring the signals of DMPO-SO_4_ adducts and DMPO-OH adducts, respectively. As shown in Fig. 13, the special hyperfine coupling constants (*a*(N) 1.49 mT, *a*(H) 1.49 mT, obtained by simulation) are completely consistent with those of DMPO-OH.^49^ Moreover, the special hyperfine coupling constants of DMPO-SO_4_ (*a*(N) 1.38 mT, *a*(H) 1.02 mT, *a*(H) 0.14 mT, *a*(H) 0.08 mT) were obtained by simulation from the spectra.^50^ All the results further confirmed that both SO_4_˙^−^ and ˙OH were generated in the δ-FeOOH/PMS system.

### 3.6. Possible activation mechanism

It has been reported that the hydroxyl groups on the surface of the metal oxide play an important role in the heterogeneous oxidation reaction.^51^ PMS can combine with the metal oxide through the surface hydroxyl groups and then undergo a redox reaction with the surface metal of oxide to produce the sulfate radical. Moreover, the oxidation state on the surface metal will consistently change with the surface hydroxyl groups.^52–54^ Thus, the *in situ* spectroscopic analysis could detect the intermediates related to PMS decomposition on the surface of metal oxide.

The *in situ* characterization of δ-FeOOH surface during catalytic decomposition of PMS was conducted *via* ATR-FTIR. As shown in Fig. 12, in the PMS solution alone, PMS had two IR bands at 1103 cm^−1^ and 1249 cm^−1^,^55^ which originated from S–O of either HSO_5_^−^ or SO_4_^2−^. However, the intensity of 1249 cm^−1^ had a significant decline when PMS was mixed with δ-FeOOH. Thus, the band at 1249 cm^−1^ is related to HSO_5_^−^. The intensity decline implied the decomposition of PMS on the oxide surface. Moreover, a red-shift by 19 cm^−1^ of S–O crest at 1249 cm^−1^ occurred with the addition of δ-FeOOH as compared to the case of the pure PMS solution. This demonstrates that –OH in HSO_5_^−^ attracts more electron density from the near S–O bond making it weaker;^53^ this may indicate that the surface metal captures electron from –OH, and an increase in the electron attraction from neighboring S–O leads to the generation of a sulfate radical.

**Fig. 14:**
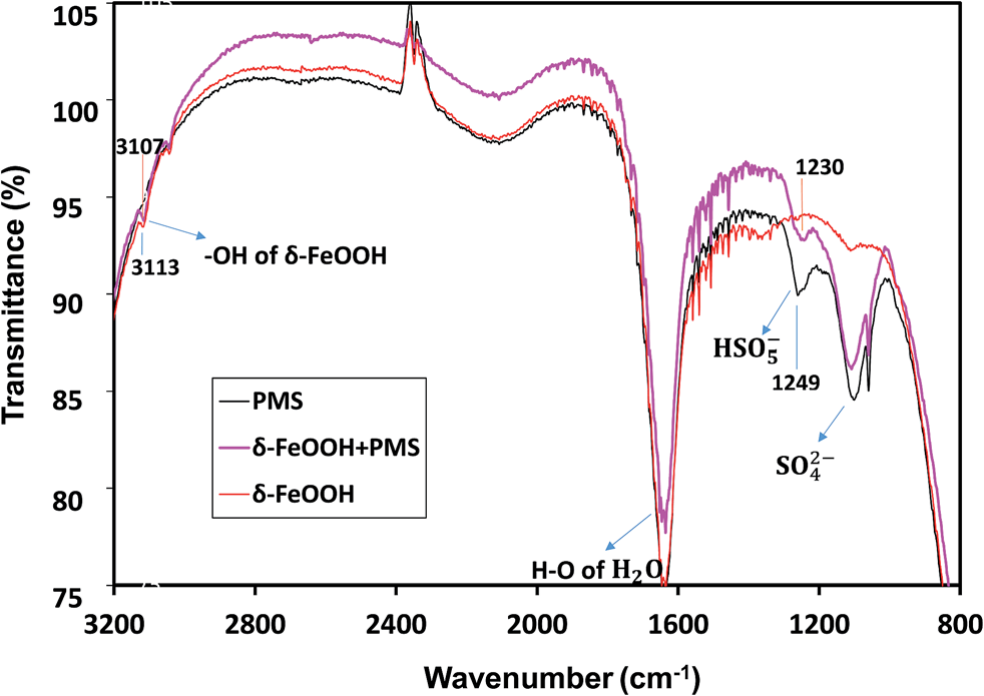
ATR-FTIR spectra of PMS solution, the δ-FeOOH suspension, and the δ-FeOOH particles in the PMS solution. Conditions: pH 5 and *t* = 25 ± 1 °C. (The inverse peaks are likely to be owned by the attrite Ge crystal of ATR attachment.)

According to Zhang *et al.*, the stretching vibration of surface hydroxyl is around 3100 cm^−1^.^56^ In Fig. 14, there is an intense peak at 3113 cm^−1^, which indicates the presence of surface –OH groups on δ-FeOOH. In the presence of HSO_5_^−^, this band was red-shifted by 6 cm^−1^. It is a symbol for the replacement or complexation of the surface –OH groups by HSO_5_^−^;^53^ this reveals the formation of a complex between HSO_5_^−^ and metal oxide, and HSO_5_^−^ loses an electron to the surface Fe(iii) to generate SO_5_^−^˙.

Based on the analysis of the obtained results, the possible activation mechanism of PMS by δ-FeOOH was proposed. PMS initially conjuncts with surface –OH of FeOOH (5). Then, SO_5_^−^˙ is generated, whereas ≡Fe(iii) is reduced to ≡Fe(ii), and the generated ≡Fe(ii) can form a complex with HSO_5_^−^(6). Next, ≡Fe(ii) can be oxidized to ≡Fe(iii); this releases SO_4_^−^˙ into the solution (7).^57^ Finally, SO_4_^−^˙ can react with OH^−^ to produce ˙OH (8) or directly attack AO7 (9), both of which are responsible for AO7 degradation (eqn (5)).^53^

5 ≡Fe(iii)–OH + HSO_5_^−^ → ≡Fe(iii)–(OH)SO_4_ + OH^−^

6 ≡Fe(iii)–(OH)SO_4_ + HSO_5_^−^ → ≡Fe(ii)–˙OOSO_3_^−^ + SO_5_^−^˙ + H^+^

7 ≡Fe(ii)–˙OOSO_3_^−^ + H_2_O → ≡Fe(iii)–OH + SO_4_^−^˙ + OH^−^

8 SO_4_^−^˙ + OH^−^ → ˙OH + SO_4_^2−^

9 AO7 + SO_4_^−^˙/˙OH → intermediate → CO_2_ + H_2_O

**Fig. 15:**
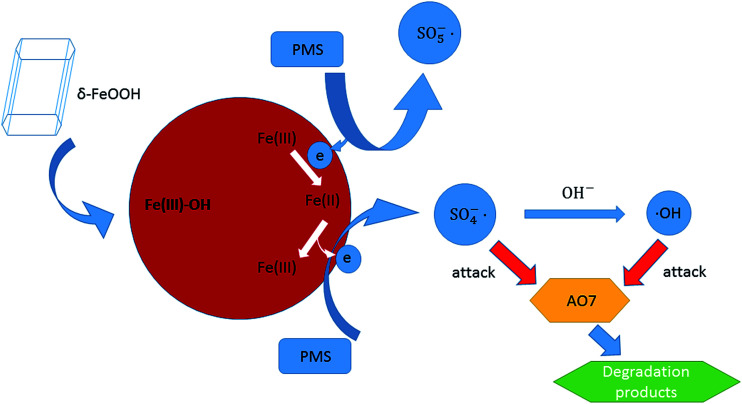
Speculated activation mechanism in the δ-FeOOH/PMS process.

The produced radicals, including SO_4_^−^˙ and ˙OH, can then attack the chromophore of AO7. All the processes are radical reactions. The proposed activation mechanism of the δ-FeOOH/PMS process can be described as shown in Fig. 15.

## 4. Conclusion

In this study, FeOOH with four different crystalline phases (α, β, γ, and δ) was synthesized and characterized by XRD, N_2_ adsorption/desorption, TEM, HRTEM, and other simple methods. Then, they were used as PMS activators for the degradation of AO7. According to the tests, δ-FeOOH was confirmed as a potential catalyst due to its much higher efficiency for PMS activation than others. The catalyst showed stability in element valences and catalytic activity during successive repeated reactions. The degradation reaction was a radical reaction, and both SO_4_^−^˙ and ˙OH were suggested as radical species in the PMS/δ-FeOOH system. The catalytic mechanism was proposed with the aid of the *in situ* ATR-FTIR analysis and XPS spectra. These results reveal that δ-FeOOH is an effective, environmentally friendly, and low cost catalyst for the efficient generation of sulfate radicals and hydroxyl radicals from PMS to degrade organic pollutants. They own a great potential in the advanced oxidative treatment of industrial wastewater and other contaminated water.

## Conflicts of interest

There are no conflicts to declare.

## Supplementary Material

RA-008-C7RA12615H-s001
