# Supplementary material for: Synthesis of different crystallographic FeOOH catalysts for peroxymonosulfate activation towards organic matter degradation
Source: RSC Adv. 2018 Feb 14;8(13):7269–79. doi: 10.1039/c7ra12615h (PMC9078432; doi:10.1039/c7ra12615h)
Supplement: RA-008-C7RA12615H-s001 [file RA-008-C7RA12615H-s001.pdf]

Electronic Supplementary Material (ESI) for RSC Advances.
This journal is © The Royal Society of Chemistry 2018

Synthesis of Different Crystallographic FeOOH for Peroxymonosulfate Activation towards organic matters degradation

Junyu Fan, [1] Zhaoxia Ding, [1] Zhiwei Zhao, [2,*] Jie Liu [1,*]

1. Department of Military Facilities, Army Logistics University, Chongqing, 401311, China

2. Key laboratory of the Three Gorges Reservoir Region's Eco-Environment, State Ministry of Education, Chongqing University, Chongqing, 400045, China

Corresponding Author

Jie Liu: Tel.: + 86 (23) 8673 0631, e-mail: liujiely@hotmail.com

Zhiwei Zhao:e-mail:zhaozhiweihit@gmail.com

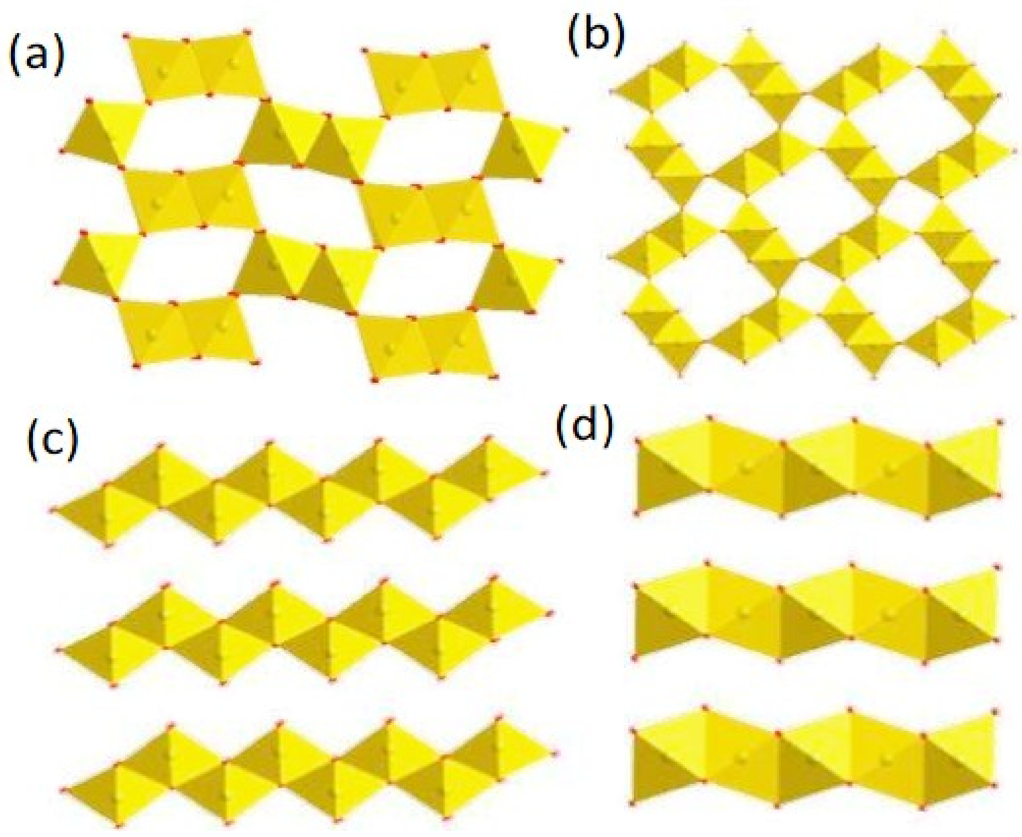

Fig. S1 Structures of the FeOOH polymorphs. (a) α-FeOOH (goethite), (b) β-FeOOH(akaganeite), (c) γ-FeOOH(lepidocrocite) and (d) δ-FeOOH(feroxyhyte).

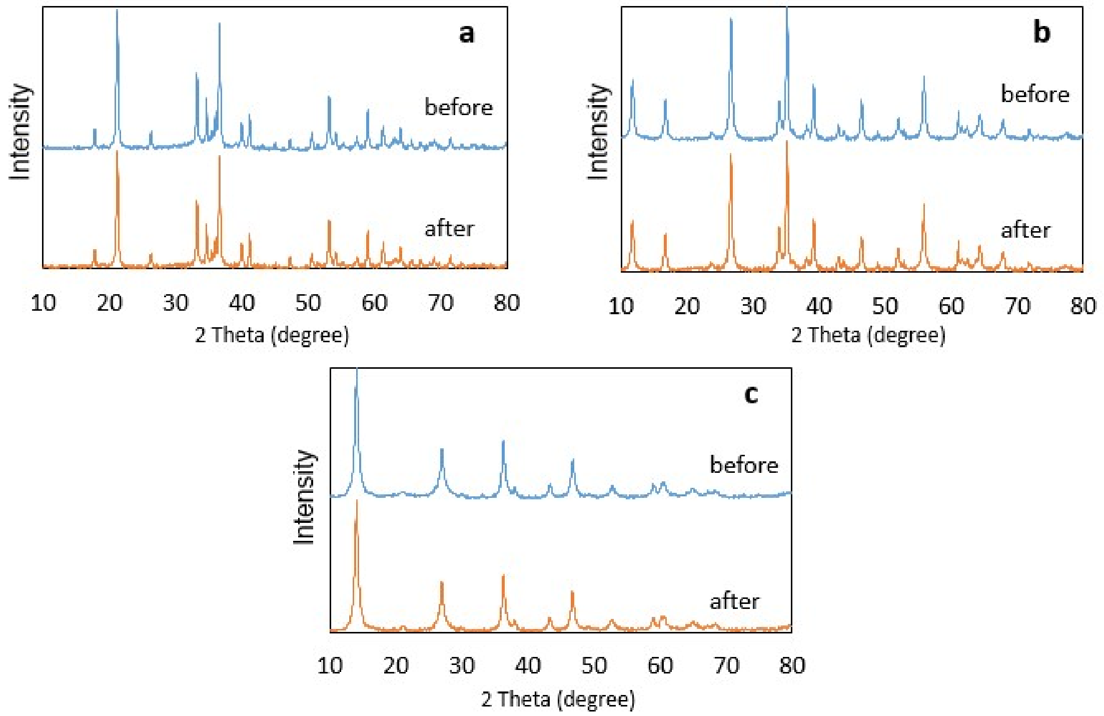

Fig. S2 XRD patterns of synthesized α-FeOOH, β-FeOOH and γ-FeOOH before and after used 6 times in PMS/δ-FeOOH/AO7 system.

Table S1. Decolorzation efficiency of AO7 with catalyst of α-FeOOH, β-FeOOH and γ-FeOOH during six consecutive cycles.

Time of reusability	Decolorzation efficiency (%)		
	α-FeOOH	β-FeOOH	γ-FeOOH
1	42%	24.9%	29.5%
2	40.4%	24.5%	27.9%
3	39.2%	24.2%	27.1%
4	38.7%	24.2%	26.5%
5	38.1%	23.9%	26.0%
6	37.6%	23.7%	25.6%

Table S2. Metal ions leaching amount of α-FeOOH, β-FeOOH and γ-FeOOH during six consecutive cycles.

Time of reusability	Iron leaching concentration (μg L^{-1})		
	α-FeOOH	β-FeOOH	γ-FeOOH
1	5.11	5.24	4.85
2	4.98	4.92	4.82
3	4.76	4.83	4.39
4	4.32	4.43	4.51
5	3.94	4.65	4.03
6	3.49	4.29	3.87
